# Limited Utility of Existing Hearing Loss Panels in the Assessment of Early‐Onset, Bilateral Meniere's Disease

**DOI:** 10.1002/oto2.70180

**Published:** 2025-12-09

**Authors:** Keshav V. Shah, Christian Jung, Daniel Talian, Sherrie Davis, Douglas J. Epstein, Michael J. Ruckenstein, Tiffany P. Hwa

**Affiliations:** ^1^ Department of Otorhinolaryngology–Head and Neck Surgery University of Pennsylvania Philadelphia Pennsylvania USA; ^2^ Department of Genetics University of Pennsylvania Philadelphia Pennsylvania USA

**Keywords:** endolymphatic hydrops, genetic testing, genetics, Meniere's disease, sensorineural hearing loss

## Abstract

**Objective:**

While the etiology of Meniere's disease (MD) is likely multifactorial, genetics are thought to play a role. Several previous studies have yielded inconclusive results, potentially due to phenotypic uncertainty and variable diagnostic criteria. To explore potential genetic bases in a more rigorous context, we assessed the clinical predictors and diagnostic yield of current hearing loss panels in a highly curated cohort of patients with bilateral and/or early‐onset MD.

**Study Design:**

Retrospective cohort study.

**Setting:**

Multidisciplinary tertiary care hearing loss genetics clinic.

**Methods:**

Data from clinical notes, audiograms, and genetic reports of adult patients diagnosed with bilateral and/or early‐onset (<40 years) MD from October 2019 to June 2025 were analyzed with logistic regression and summary statistics to determine predictive factors and diagnostic yields of existing genetic panels.

**Results:**

Of the 37 patients analyzed (mean age 47.7 + 14.5 years, 54% male), 24 (64.8%) had early‐onset MD, 22 (59.5%) had bilateral MD, and 9 (24.3%) had both. Moderately severe to profound hearing loss prior to 65 was significantly associated with pathogenic or likely pathogenic variants (PLPV) (OR 8.98 [1.17, 101]; *P* = .046). No significant predictors were found for definitive diagnosis, plausible diagnosis, or negative panels. Eight (22%) patients had a PLPV detected on their hearing loss panel, with 0 definitive diagnoses, 3 (8.1%) plausible diagnoses (*MYO15A*, *SLC17A8*, *P2RX2*), and 6 (16%) completely negative panels.

**Conclusions:**

Current hearing loss panels show limited diagnostic utility for MD. Future research should prioritize whole genome sequencing to identify novel MD‐associated loci and provide guidance to patients.

Meniere's disease (MD) is a complex inner ear disorder characterized by the clinical triad of episodic vertigo, fluctuating sensorineural hearing loss, and tinnitus, often accompanied by aural fullness.^1^, ^2^, ^3^ The underlying pathophysiology is thought to involve endolymphatic hydrops, or an abnormal accumulation of endolymph within the inner ear, but the precise mechanisms leading to this condition remain incompletely understood.^3^, ^4^, ^5^, ^6^ Nevertheless, while the etiology of MD remains unclear and is likely multifactorial, mounting evidence suggests a genetic component, particularly in patients with early‐onset disease, those with bilateral involvement, and families with multiple affected members.^7^, ^8^, ^9^

A significant challenge in managing MD lies in its status as a diagnosis of exclusion, requiring clinicians to rule out other causes of similar symptoms, including vestibular migraine, acoustic neuroma, and autoimmune inner ear disease.^10^, ^11^ This diagnostic process can lead to substantial delays in establishing the correct diagnosis and initiating appropriate treatment, during which patients may experience progressive hearing loss and continued vestibular episodes.^12^, ^13^ As genetics and genomics have started to revolutionize disease understanding and treatment approaches, there is growing interest in leveraging genetic information to identify patients at risk for developing MD.^14^, ^15^ Early identification of genetic predisposition could potentially enable implementation of preventative measures or earlier intervention strategies in what can otherwise be a debilitating and progressive condition.^16^, ^17^, ^18^

Several candidate genes have been implicated in MD pathogenesis, including those involved in ion homeostasis, immune regulation, and inner ear development.^7^, ^19^, ^20^ Many of these genes, such as *OTOG*, *TECTA*, *FAM136A*, and *DTNA*, have been discovered through family studies or sporadic cases with multiple affected individuals carrying variants in the same loci.^21^, ^22^, ^23^ However, there is still much to learn about the genetic architecture of the disease.^24^ Prior genome‐wide association studies (GWAS) have greatly expanded the repertoire of genes potentially linked to MD, but other studies have yielded inconclusive results regarding the underlying pathophysiologic mechanisms of MD, potentially due to phenotypic uncertainty and variable diagnostic criteria.^15^, ^20^, ^25^ Additionally, current commercially available hearing loss panels have been developed primarily based on genes associated with congenital and childhood hearing loss, with variable coverage of genes potentially relevant to MD.^22^, ^26^, ^27^ In the same vein, limited data exist regarding the diagnostic efficacy of these panels in MD patients, as most validation studies have focused on pediatric populations.^7^, ^28^, ^29^

Given its presumed distinct molecular basis from other etiologies of hearing loss, we hypothesized that current hearing loss panels may not be optimal tools for detecting genetic variants associated with MD. To examine this further, this study aimed to identify predictive variables and assess the diagnostic yield of commercially available hearing loss panels in patients with bilateral and/or early‐onset MD, providing the first controlled evaluation of genetic testing utility in this population. We chose this highly curated cohort of MD patients with well‐defined clinical phenotypes as they are most suggestive of an underlying genetic component, thereby maximizing the potential for existing panels to result in clinically meaningful outcomes.

## Methods

### Study Design, Setting, and Sample

A retrospective cohort study was conducted involving adult patients who were diagnosed with early‐onset (<40 years) and/or bilateral MD at a multidisciplinary tertiary care hearing loss genetics clinic from October 2019 to June 2025. The diagnosis of MD was made in all patients who met AAO‐HNS clinical diagnostic criteria and had no other explanatory etiology (eg, vestibular migraine, benign paroxysmal positional vertigo, multiple sclerosis, infection, etc.), and it was verified by a neurotologist.^10^ Those with bilateral MD met criteria on both sides. Given the focus of our clinic, every patient we saw was offered commercial genetic testing after extensive counseling regarding the types of hearing loss (syndromic versus non‐syndromic), benefits and limitations of genetic testing, types of results they may receive, commercial panels available to them, and protections afforded to them by federal law, among other things. Currently, we primarily offer patients Invitae's Comprehensive Deafness Panel, but we have also historically offered panels from GeneDx and Blueprint Genetics depending on insurance coverage, out‐of‐pocket cost to the patient, and availability.^30^, ^31^, ^32^ Buccal swabs were collected from consenting patients for all panels. Clinical notes, audiograms, and genetic panel reports from the medical records of included patients were reviewed. Patients were excluded if they did not have a documented diagnosis of MD in their electronic medical record, even if they had appropriate symptomology; if they had hearing loss attributable to other etiologies; and if they declined genetic testing. This study was approved by the University of Pennsylvania Institutional Review Board (project number 856926) and adheres to the “Strengthening the Reporting of Observational Studies in Epidemiology” framework.^33^

### Study Variables

Study variables included demographic information, audiologic and otologic history, audiometric information, family history of MD and hearing loss, and results of genetic testing. Patient age and sex assigned at birth were collected. Audiologic and otologic history included age of MD symptom onset, laterality of MD, and presence of additional otovestibular symptoms. Audiometric information included hearing loss severity based on four‐frequency PTA (500 Hz, 1 kHz, 2 kHz, and 4 kHz) and symmetry. Genetic testing results included the identification of pathogenic or likely pathogenic variants (PLPV) and variants of uncertain significance (VUS) based on definitions established by the American College of Medical Genetics and Genomics.^34^ It also included the presence of completely negative tests (patients for which no PLPV or VUS was identified), definitive diagnoses, and plausible diagnoses (patients with combinations of PLPV and/or VUS that, based on similar variants, literature review, and/or reported in silico modeling, can explain the observed hearing loss, in addition to those with definitive diagnoses).

### Study Outcomes

The primary outcome of interest was the identification of predictive associations between clinically significant variables and genetic testing results. The secondary outcomes of interest were the diagnostic yield of currently available genetic panels, including the proportion of panels with identified PLPV and VUS, definitive and plausible diagnoses, and completely negative results, and all named variants themselves.

### Statistical Methods

Summary statistics were used to describe our final cohort and determine the distribution of genetic test results. Logistic regression was used to conduct univariate analyses. Significance was set at *P* < .05, *P*‐values were 2‐sided, and 95% confidence intervals (CI) were constructed where appropriate. Multivariable logistic regression was utilized to determine variables that were predictive of genetic testing results. Statistical analyses were performed using R version 4.3.1 (R Project for Statistical Computing) via RStudio version 2025.05.1 + 513 (Posit).

## Results

### Demographics and Clinical Characteristics

Of the 37 patients included in the final cohort, 24 (64.8%) had early‐onset MD, 22 (59.5%) had bilateral MD, and 9 (24.3%) had both. The mean age of patients at presentation in the cohort was 47.7 years old (SD: 14.5 years), with a slight male majority (54.1%). Almost all patients (81.1%) had hearing loss disproportionate to age, with 9 (24.3%) having moderately severe to profound hearing loss in their worse ear. Although 26 (70.3%) patients had bilateral hearing loss, only 9 (24.3%) had symmetric hearing loss. Additional otovestibular symptoms were noted in virtually all patients (97.3%), with tinnitus being the most common (94.6%). Additional information about the patient cohort can be found in Table 1.

**Table 1:** Demographic and Clinical Characteristics of Patient Cohort

Variable	n (%); mean (SD)
Mean age at presentation (years)	47.7 (14.5)
Male	19 (54)
Early‐onset MD	24 (65)
Bilateral MD	22 (59)
Moderately severe or worse HL	7 (19)
Bilateral HL	26 (70)
Symmetric HL	9 (24)
Additional otovestibular symptoms	36 (97)
Tinnitus	35 (95)
Aural fullness	26 (70)
Otalgia	3 (8.1)
Decade of life of first MD symptom	
1	1 (2.7)
2	2 (5.4)
3	8 (22)
4	13 (35)
5	5 (14)
6	8 (22)
Family History	
First‐degree relative with HL	4 (10.8)
Second‐degree relative with HL	5 (14)
Any family history of MD	3 (8.1)

Abbreviations: HL, hearing loss; MD, Meniere's disease.

### Predictive Associations

On univariate analysis of all previously mentioned study variables, moderately severe to profound hearing loss before the age of 65 was significantly associated with PLPV (OR 8.33 [1.38, 59.1]; *P* = .023). Patients with a first‐degree relative with hearing loss had a higher, non‐significant likelihood for plausible diagnosis (OR 6.83 [0.74, 55.8]; *P* = .068). No clinical variables reached significance for definitive diagnosis or negative tests.

Moderately severe‐to‐profound hearing loss remained significant for PLPV (OR 8.98 [1.17, 101]; *P* = .046) in a multivariable regression model that also included the following variables: MD diagnosis at <40 years old, bilateral MD, first‐degree relative with hearing loss. Multivariable models were not constructed for definitive diagnosis, plausible diagnosis, or negative panels given the lack of statistically significant findings on univariate analysis and concerns with small sample size. Subgroup analysis was not performed for patients with bilateral, early‐onset MD given the small sample size.

### Diagnostic Yield of Commercially Available Panels

Of the 37 patients analyzed, 8 (22%) had a PLPV detected on their hearing loss panel, with 0 definitive diagnoses, 3 (8.1%) plausible diagnoses (*MYO15A*, *SLC17A8*, *P2RX2*), and 6 (16%) completely negative panels. The majority of patients (64.9%) only tested positive for a VUS. In patients with both bilateral and early‐onset symptoms, 3 (33%) had a PLPV and 4 (44%) had only a VUS, with 1 (11%) plausible diagnosis (*SLC17A8*) and 1 (11%) completely negative panel. A complete list of genetic testing results, including identified variants with P and C dot notation, can be found in Table 2.

**Table 2:** Complete Results of Genetic Testing of Patient Cohort

#	Age at presentation	Sex	Early onset	Bilateral MD	Worst HL severity	Bilateral HL	Symmetric HL	Tinnitus	Aural fullness	Otalgia	PLPV	VUS	Plausible diagnosis	Negative test
1	22	M	✓	✓	Moderately severe	✓		✓	✓		‐‐	TSPEAR (c.1339 G>A, p.Asp447Asn)	‐‐	‐‐
2	25	F	✓	✓	Profound	✓		✓	✓		‐‐	SLC17A8 (c.200 G>A, p.C67Y); SLC17A8 (c.200 G>A, p.C67Y); PCDH15 (c.3857 T>A, p.V1286E); USH1G (c.231C>G, p.H77Q)	✓	‐‐
3	27	F	✓		Moderately severe		✓	✓			‐‐	TMPR223 (c.1042G>A, p.Asp348Asn)	‐‐	‐‐
4	28	F	✓	✓	Mild	✓	✓	✓	✓	✓	‐‐	ESPN (c.950A>G, p.His317Arg); SLITRK6 (c.1591 T>C, p.Trp531Arg); ESRRB (c.244 G>C, p.Gly82Arg), CDH23 (c.5146 C>A, p.Gln1716Lys), ADGRV1 (c.853 C>G, p.Arg285Gly)	‐‐	‐‐
5	29	M	✓		Moderately severe		✓	✓			GJB2 (c.239A>C, p.Gln80Pro)	TRIOBP (c.488A>C, p.Glu163Ala)	‐‐	‐‐
6	33	M	✓	✓	Moderately severe	✓		✓	✓		‐‐	OTOF (c.5405C>T, p.Ala1802Val)	‐‐	‐‐
7	33	M	✓	✓	Moderately severe	✓	✓	✓	✓		‐‐	‐‐	‐‐	✓
8	33	M	✓		Moderate			✓	✓		‐‐	BDP1 (c.3466G>A, p.E1156K)	‐‐	‐‐
9	35	F	✓		Moderate			✓	✓		‐‐	‐‐	‐‐	✓
10	36	F	✓		Moderate			✓	✓		‐‐	‐‐	‐‐	✓
11	37	M	✓		Moderate			✓	✓		‐‐	USH1G (c.1158G>T, p.Glu386Asp)	‐‐	‐‐
12	39	M	✓		Severe	✓		✓	✓		ALMS1 (c.9754C>T, p.Gln3252*)	S1PR2 (c.632G>A, p.Arg211His); CLDN14 (c.286G>A, p.Ala96Thr)	‐‐	‐‐
13	40	M	✓		Moderately severe		✓	✓			‐‐	FGFR2 (c.1531G>T, p.Ala511Ser)	‐‐	‐‐
14	40	F	✓		Severe			✓	✓		‐‐	KCNJ10 (c.1123C>T, p.Arg375Cys); WBP2 (c.172A>T, p.Ile58Phe); ARSG (c.430G>A, p.Gly144Ser); CHD7 (c.3121T>G, p.Leu1041Val)	‐‐	‐‐
15	41	F	✓		Severe			✓	✓		‐‐	TSPEAR (c.1583A>C, p.Asp528Ala)	‐‐	‐‐
16	43	F	✓		Severe			✓			‐‐	OTOF (c.505C>G, p.Arg169Gly); OTOG (c.6572T>C, p.Val2191Ala)	‐‐	‐‐
17	45	M	✓		Mild	✓	✓				‐‐	MITF (c.1238G>A, p.Arg413Gln)	‐‐	‐‐
18	46	F	✓		Severe	✓		✓	✓		GJB2 (p.35del, c.Gly12Valfs*2)	‐‐	‐‐	‐‐
19	46	F	✓	✓	Severe	✓		✓	✓		‐‐	‐‐	‐‐	✓
20	47	F		✓	Moderately severe	✓	✓	✓	✓	✓	‐‐	COL11A2 (c.2519G>A, p.Arg840Gln)	‐‐	‐‐
21	50	F		✓	Profound	✓		✓	✓		‐‐	OTOF (c.4435G>A, p.Gly1479Ser)	‐‐	‐‐
22	50	M	✓		Moderate			✓			‐‐	MYO15A (c.8644G>A, p.Asp2882Asn); MYO15A (c.9785G>C, p.Arg3262Pro)	✓	‐‐
23	55	M	✓		Profound	✓		✓			‐‐	OTOG (c.5416C>T, p.Arg1806Cys)	‐‐	‐‐
24	57	M		✓	Moderate	✓	✓	✓	✓		‐‐	‐‐	‐‐	✓
25	58	M	✓	✓	Profound	✓		✓	✓		TYR (c.613c>A, p.Pro205Thr); TECTA (c.1048C>T, p.Arg350*)	‐‐	‐‐	‐‐
26	58	M		✓	Moderate	✓		✓	✓	✓	‐‐	EPSPN (c.1699_1701dup, p.Pro567dup); HARS1 (c.1159G>A, p.Glu387Lys)	‐‐	‐‐
27	58	M		✓	Profound	✓	✓	✓			‐‐	P2RX2 (c.381+2T>C, p.?)	✓	‐‐
28	59	M		✓	Moderately severe	✓	✓	✓			‐‐	LRP2 (c.2342A>G, p.Asn781Ser); PEX19 (c.896T>C, p.Met299Thr)	‐‐	‐‐
29	64	M		✓	Profound	✓		✓			GJB2 (c.35delG, p.Gly12ValfsX2)	TECTA (c.4802A>G, p.Asn1601Ser)	‐‐	‐‐
30	64	M		✓	Moderately severe	✓		✓				MYO7A (c.2122A>G, p.Met708Val)	‐‐	‐‐
31	64	F		✓	Moderately severe	✓		✓				ADGRV1 (c.12552G>T, p.Lys4184Phe)	‐‐	‐‐
32	65	M		✓	Profound	✓		✓	✓		COLL11A1 (c.2292_2295dupAAAG, p.Gly766LysfsX13)	DIAPH1 (c.‐3G>C, p.?); PMP22 (c.353C>T, p.Thr118Met); WFS1 (c.353C>T, p.Thr118Met), ALMS1 (c.6891A>C, p.Gln2297His); PTPRQ (c.1627A>G, p.Met543Val)	‐‐	‐‐
33	65	F	✓	✓	Profound	✓		✓	✓			DNMT1 (c.1008G>A, silent); MYO7A (c.1757A>G, p.Asn586Ser)	‐‐	‐‐
34	67	M		✓	Moderately severe	✓		✓			‐‐	‐‐	‐‐	✓
35	67	F	✓	✓	Profound	✓	✓	✓	✓		OTOA (deletion exons 1‐19)	‐‐	‐‐	‐‐
36	68	F		✓	Moderately severe	✓	✓		✓			SCP2 (c.884C>A, p.Thr295Lys)	‐‐	‐‐
37	71	F		✓	Profound	✓		✓			STRC (c.(3794 + 1_3795‐1)_(*1_?)del, p.exons19‐29)	‐‐	‐‐	‐‐

## Discussion

This study represents the first controlled evaluation of commercially available hearing loss panels in patients with MD, focusing on a carefully selected cohort enriched for genetic susceptibility through early‐onset and/or bilateral disease criteria. Our results demonstrate that current hearing loss panels have limited diagnostic utility in MD patients, with only 22% of patients tested harboring PLPV and no definitive diagnoses established. The majority (65%) of patients received only VUS, highlighting a significant gap between the clinical need and current genetic testing capabilities in this population.

Among the clinical variables examined, moderately severe to profound hearing loss prior to age 65 emerged as the sole significant predictor of positive genetic testing results on both univariate and multivariate analysis. However, this finding may reflect the largely hearing loss‐centric design of current genetic panels rather than MD‐specific genetic architecture. Current panels were developed to detect variants associated with hearing loss broadly, and this finding aligns with previous studies demonstrating that more severe hearing loss and positive family history are predictive of positive results across various etiologies.^35^, ^36^ Notably, characteristics more specific to MD pathophysiology—including otovestibular symptoms, age of MD symptom onset and diagnosis, and bilateral MD—failed to demonstrate predictive value, suggesting that the genetic underpinnings of MD may involve distinct pathways not adequately captured by current hearing loss‐focused gene panels.

This disconnect between hearing loss and MD genetics may explain why even our curated cohort yielded disappointing results. MD likely involves genes related to vestibular function, endolymphatic homeostasis, or immune regulation that are not traditionally considered “hearing loss genes” and therefore absent from current panels.^7^, ^20^ The complex clinical presentation of MD involving both auditory and vestibular symptoms with significant phenotype heterogeneity likely reflects an equally complex genetic architecture that cannot be captured by targeted gene panels.

The predominance of VUS results and absence of definitive diagnoses in our cohort further underscores this fundamental mismatch. Commercially available hearing loss panels were primarily developed and validated using pediatric populations with congenital and early‐onset hearing loss, conditions with well‐characterized monogenic etiologies and high penetrance variants.^37^, ^38^ In contrast, MD likely represents a more genetically heterogenous condition with complex inheritance patterns and incomplete penetrance.^39^ This suggests that substantial genetic territory remains unexplored in MD, challenging the routine use of current hearing loss panels in these patients. In clinical practice, the high frequency of VUS also provides limited actionable information while potentially causing anxiety and uncertainty for patients and families.

Given the absence of definitive or plausible diagnoses in most patients, we advocate for whole genome sequencing (WGS) as the preferred modality for genetic assessment of MD. This approach offers several advantages, including comprehensive coverage of both known and novel genetic loci, ability to detect structural variants and copy number variations not captured by targeted panels, and potential for reanalysis as new MD‐associated genes are discovered.^40^, ^41^ WGS may be particularly valuable in identifying rare variants in genes not traditionally associated with hearing loss but potentially relevant to inner ear physiology, such as those involved in ion transport, immune function, or vestibular development. Of note, there was one reported VUS—*OTOG* (c.5416C>T, p.Arg1806Cys)—that results in a missense mutation that does not seem to be reported in the literature. Given its potential impact on protein structure and the fact that *OTOG* is a candidate gene for MD, this result does demonstrate the utility of further exploring genetic testing in this highly curated cohort. However, most genetic variation for MD appears to be in noncoding regions.^39^, ^42^, ^43^ As such, the WGS approach may be better suited for the discovery of new genetic loci specific to MD pathogenesis, advancing our understanding of disease mechanisms and potentially identifying novel therapeutic targets.

Some limitations must be acknowledged when interpreting these findings. Our relatively small sample size, while representative of the specialized nature of tertiary care MD populations, limits statistical power and generalizability of results. Our sample also demonstrated a male skew rather than the female preponderance typically associated with MD, which may be a byproduct of our small sample size or an instance of referral bias (eg, males are more likely to be referred due to a perceived “atypical” presentation, males more likely to seek care or subspecialty referral, etc.). The paucity of meaningful genetic panel results further constrains our ability to identify robust predictive factors or draw definitive conclusions about the utility of genetic testing. The evolving nature of variant interpretation means that some VUS identified in our cohort may be reclassified as pathogenic or benign with additional evidence, potentially altering our conclusions over time. Because of these reasons, it is key to focus on expanding genetic discovery efforts through larger‐scale GWAS and WGS in well‐phenotyped MD cohorts. Such studies will be essential for developing MD‐specific genetic testing approaches that can provide meaningful diagnostic and prognostic information for patients and families affected by this challenging condition.

## Conclusion

Current hearing loss panels demonstrate limited diagnostic utility in patients with MD, with few predictive factors, high proportion of variants of uncertain significance, and no definitive diagnoses even in our cohort enriched for genetic susceptibility. Future research should prioritize whole‐genome sequencing approaches to identify novel MD‐associated genetic loci and develop disease‐specific testing strategies that can provide meaningful clinical guidance for this complex otologic condition.

## Author Contributions

**Keshav V. Shah**, design, conduct, analysis, writing, editing, presentation of research; **Christian Jung**, writing, editing; **Daniel Talian**, editing; **Sherrie Davis**, editing; **Douglas J. Epstein**, editing; **Michael J. Ruckenstein**, editing; **Tiffany P. Hwa**, design, editing.

## Disclosures

### Competing interests

Tiffany P. Hwa, is a grant recipient from Cochlear Corporation and serves as a consultant on the advisory board of Amgen. No conflicts of interest were declared for the remaining authors.

### Funding source

Funding sources for this study were the Gates Foundation, TGI, and the Schnabel family.
